# Efficacy and safety of acute-phase immunotherapy for adult autoimmune encephalitis: a systematic review and network meta-analysis

**DOI:** 10.3389/fimmu.2026.1807436

**Published:** 2026-10-07

**Authors:** Yan Deng, Yun Cai, Xin Yang, Lihua Xu, Yanan Chen, Na Liu, Weiying Di

**Affiliations:** Department of Neurology, Affiliated Hospital of Hebei University, Baoding, Hebei, China

**Keywords:** acute-phase immunotherapy, autoimmune encephalitis, Bayesian network meta-analysis, efgartigimod, immunoadsorption

## Abstract

**Background:**

Autoimmune encephalitis (AE) is an immune-mediated inflammatory disorder of the central nervous system associated with substantial morbidity. Acute-phase immunotherapy is crucial for improving outcomes, yet the comparative efficacy and safety of available regimens remain unclear. We therefore performed a Bayesian network meta-analysis to compare acute-phase immunotherapeutic strategies for AE.

**Methods:**

PubMed, Embase, Web of Science, and the Cochrane Library were searched from inception to October 2025 for studies evaluating acute-phase immunotherapy in AE. A Bayesian network meta-analysis was conducted. The primary outcome was change in the modified Rankin Scale (mRS) after treatment; adverse events were secondary outcomes. Effects were expressed as mean differences (MD) and risk ratios (RRs) with 95% credible intervals (CrIs). The surface under the cumulative ranking curve (SUCRA) was used to rank interventions.

**Results:**

Eleven studies (1,359 patients) were included, evaluating seven strategies: intravenous methylprednisolone (IVMP), intravenous immunoglobulin (IVIG), IVMP+IVIG, IVMP+plasma exchange (PE), IVMP+IVIG+PE, IVMP+immunoadsorption (IA), and IVMP+efgartigimod (EFG). For efficacy, IVMP+EFG ranked highest in mRS improvement among the included regimens (MD = −0.99, 95% CrI −1.30 to −0.68; SUCRA = 95%), followed by IVMP+IA (MD = −0.87; SUCRA = 88%). IVMP+PE ranked lowest (MD = 0.32; SUCRA = 0.2%). For safety, IVIG monotherapy had the lowest adverse-event risk (RR = 0.51, 95% CrI 0.24 to 0.97), whereas IVMP+PE had the highest risk (RR = 3.10, 95% CrI 0.92 to 9.60).

**Conclusion:**

Acute-phase immunotherapy for AE should be individualized by disease severity and antibody subtype. Consistent directional findings were observed in the anti-NMDAR subgroup and, separately, in the subgroup with baseline mRS ≥3. IVMP+EFG and IVMP+IA ranked favorably for short-term functional improvement, although the evidence was based mainly on observational studies and small sample sizes. IVIG remained a reasonable option given its favorable safety profile.

**Systematic review registration:**

https://www.crd.york.ac.uk/PROSPERO/view/CRD420251085606, identifier CRD420251085606.

## 1 Background

Autoimmune encephalitis (AE) comprises a group of antibody-mediated inflammatory disorders of the central nervous system (CNS), accounting for approximately 10–20% of all encephalitis cases. Among these, anti–N-methyl-D-aspartate receptor (anti-NMDAR) and anti–leucine-rich glioma-inactivated 1 (anti-LGI1) encephalitis represent the more prevalent subtypes (1). AE carries a high risk of disability and may result in profound neurological sequelae, including cognitive impairment, memory deficits, epileptic seizures, and psychiatric or behavioral disturbances (2, 3). These deficits substantially compromise patients’ quality of life and impose a considerable burden on healthcare systems. Timely and appropriate immunotherapy during the acute phase is pivotal for improving neurological recovery and minimizing complications. The choice of therapeutic regimen directly influences both functional outcomes and the incidence of overall adverse events, underscoring the clinical importance of identifying effective acute-phase AE treatment strategies.

With advances in immunological therapeutics, acute management of AE has evolved beyond single-modality approaches toward a diversified framework integrating conventional and novel therapies. Traditional regimens encompass intravenous methylprednisolone (IVMP) and intravenous immunoglobulin (IVIG) as monotherapies or in combination, as well as intensified protocols such as IVMP combined with plasma exchange (PE) or IVMP+IVIG+PE (4); more recently, emerging therapies including IVMP combined with the FcRn antagonist efgartigimod (EFG) and IVMP combined with immunoadsorption (IA) have expanded the therapeutic armamentarium (5, 6). Despite accumulating evidence, findings regarding the efficacy and safety of these interventions remain inconsistent. For example, some studies suggest that IVMP+IVIG confers superior short-term responses and reduces relapse rates compared with monotherapy, whereas others report inferior neurological recovery and an increased risk of nosocomial infections with combination therapy (7, 8). These discrepancies highlight the need for a robust comparative synthesis to clarify the relative benefits and risks of available acute-phase immunotherapeutic options in AE.

In recent years, the emergence of novel therapeutic strategies has opened new avenues for the clinical management of AE. Combination regimens such as IVMP+EFG and IVMP+IA have demonstrated notable potential in antibody clearance and prognostic improvement among patients with severe disease compared with conventional monotherapy approaches (9). However, existing evidence has largely been confined to comparisons between novel therapies and traditional single-agent regimens, with a notable lack of systematic head-to-head evaluations among novel interventions themselves or between novel and traditional combination therapies. This limitation hampers a comprehensive appraisal of their relative clinical benefits and safety profiles. Moreover, the hierarchical ranking of treatment efficacy and the differential risks associated with various regimens remain insufficiently defined, resulting in a paucity of high-quality evidence to support individualized therapeutic decision-making in clinical practice (10).

This study applies a Bayesian network meta-analytic framework to systematically synthesize available clinical evidence and comprehensively compare the efficacy and safety of immunotherapeutic strategies administered during the acute phase of AE. Changes in the modified Rankin Scale (mRS) score before and after treatment were selected as the primary efficacy outcome, while the incidence of adverse events served as the safety endpoint. Treatment effects were quantified using mean differences (MDs), risk ratios (RRs), and the surface under the cumulative ranking curve (SUCRA) to delineate comparative efficacy rankings and safety characteristics across regimens. In parallel, stratified analyses were implemented according to antibody subtype and baseline disease severity. Through this integrative approach, robust and clinically actionable evidence is provided to inform individualized treatment selection in acute AE, address current therapeutic decision-making challenges, and ultimately improve patient outcomes and quality of life.

## 2 Methods

This study adhered to the Preferred Reporting Items for Systematic Reviews and Meta-Analyses (PRISMA) guidelines, prospectively registered in the PROSPERO database (Registration No. CRD420251085606).

### 2.1 Data sources

A systematic literature search was performed in PubMed, Embase, Web of Science, and the Cochrane Library, covering the period from database inception to October 31, 2025. The search strategy integrated controlled vocabulary terms (e.g., MeSH terms) and free-text keywords. Core search terms included “Autoimmune Encephalitis,” “Methylprednisolone,” “Intravenous Immunoglobulins,” and “Plasma Exchange,” along with relevant synonyms and variants. No language restrictions were applied. In addition, reference lists of eligible studies and relevant reviews were manually screened to ensure comprehensive literature coverage (The detailed search strategy is presented in **Supplementary Table S1**).

### 2.2 Study selection

Study screening was independently conducted by two investigators (Deng and Di). Titles and abstracts were initially reviewed, followed by full-text assessment to determine final eligibility. Discrepancies were resolved through discussion with a third investigator (Cai). Study selection adhered to the PICOS framework as follows: (1) Population: Adults (≥18 years) with a confirmed diagnosis of autoimmune encephalitis, irrespective of antibody subtype (including anti-NMDAR, anti-LGI1, and others). Studies primarily investigating classical paraneoplastic neurological syndromes, infectious encephalitis, or immune checkpoint inhibitor-associated encephalitis were excluded. (2) Interventions: Any single or combined immunotherapeutic regimen administered during the acute phase of AE, including but not limited to IVMP, IVIG, PE, IA, EFG, and their combinations. (3) Control: Placebo or supportive care, or alternative immunotherapeutic regimens. (4) Outcomes: The primary outcome was the change in mRS score before and after treatment; secondary outcomes included overall adverse events. (5) Study design: Randomized controlled trials (RCTs) and cohort studies. Exclusion criteria: (1) Reviews, case reports, conference abstracts, or editorial letters; (2) Studies with a sample size <10 participants; (3) Studies lacking extractable outcome data; (4) Studies primarily evaluating second-line or sequential therapies; (5) Duplicate publications based on overlapping datasets.

### 2.3 Risk of bias assessment

Risk of bias was evaluated using study design–specific instruments. RCTs were assessed with the National Heart, Lung, and Blood Institute (NHLBI) Quality Assessment Tool for Controlled Intervention Studies, which is specifically designed to systematically identify sources of bias affecting internal validity. Cohort studies were appraised using the NHLBI Quality Assessment Tool for Observational Cohort and Cross-Sectional Studies, which similarly facilitates structured evaluation of internal bias in observational research. Assessments were independently completed by two investigators, with each item scored according to predefined criteria covering population selection, intervention or exposure definition, outcome measurement, confounding control, and data completeness. Disagreements were resolved via discussion, and final judgments were consolidated by a third investigator. Studies were rated as good, fair, or poor quality according to the overall pattern of methodological limitations.

### 2.4 Data extraction

Two investigators (Deng and Di) independently extracted data from eligible studies using a standardized data extraction form. All extracted data were cross-checked to ensure accuracy and completeness. Any discrepancies were settled through discussion; when consensus could not be reached, adjudication was performed by a third senior clinical research expert. Extracted variables included: (1) Study characteristics: first author, year of publication, country, and study design; (2) Baseline patient characteristics: total sample size, age, sex distribution, antibody subtype, and baseline mRS score; (3) Intervention details: immunotherapeutic regimens (monotherapy, dual therapy, or triple therapy and specific drug combinations), dosage, and treatment duration; (4) Outcome data: Efficacy was evaluated based on the change in mRS score from baseline to post-treatment. Safety was assessed by the number of patients with at least one adverse event during acute treatment or follow-up, as defined per original study. This included all adverse events, regardless of severity, infection status, or attribution to the treatment. All outcome data were extracted as unadjusted study level aggregate values. Data on the interventions included in the studies are detailed in **Supplementary Table S2**.

### 2.5 Data synthesis

Statistical analyses were conducted using R version 4.5.0 with the gemtc, mvmeta, and netmeta packages. A Bayesian framework was applied to construct a network meta-analysis model. Effect measures were selected according to outcome type: continuous outcomes (mRS scores) were synthesized using MDs with 95% credible intervals (CrIs), whereas dichotomous outcomes (adverse events) were summarized using RRs with 95% CrIs. For zero-event safety arms, estimates were generated under the Bayesian binomial model with vague priors; non-estimable interventions were excluded from SUCRA ranking. Network plots were generated to visualize treatment comparisons, with nodes representing interventions and edges indicating direct comparisons; edge thickness was proportional to the number of contributing studies. For networks containing closed loops, inconsistency was assessed using the node-splitting approach, with a P value >0.05 indicating acceptable agreement between direct and indirect evidence. Bayesian model specifications included four Markov chains, a scaling factor of 2.5, 10,000 burn-in iterations, 40,000 total iterations, and a thinning interval of one, ensuring adequate convergence and model stability. Sensitivity analyses were additionally performed in patients with anti-NMDAR encephalitis and in those with baseline mRS scores ≥3 to further examine robustness, as well as exclusion of the mixed LGI1/CASPR2 cohort. Overall treatment ranking was quantified using the SUCRA, with values closer to 1 indicating superior therapeutic performance. For the adverse-event outcome, higher SUCRA values corresponded to a lower adverse-event risk; therefore, higher values denoted a more favorable safety profile.

## 3 Results

### 3.1 Results of study selection

The PRISMA flow diagram (**Figure 1**) depicts the study selection process. An initial search retrieved 6,381 records (PubMed, n=2,831; Embase, n=3,026; Cochrane Library, n=277; Web of Science, n=247), with three additional studies identified through citation tracking. After removal of 762 duplicates, 5,619 records underwent title and abstract screening, of which 5,459 were excluded for not meeting preliminary inclusion criteria. The remaining 160 records, together with the three additional records identified through other sources (total n=163), were subjected to full-text eligibility assessment. Of these, 152 studies were excluded due to ineligible populations (n=34), inappropriate study design (n=42), irrelevant outcomes (n=31), or unsuitable interventions (n=45). This process resulted in the inclusion of 11 studies in the final analysis (11–21).

**Figure 1:**
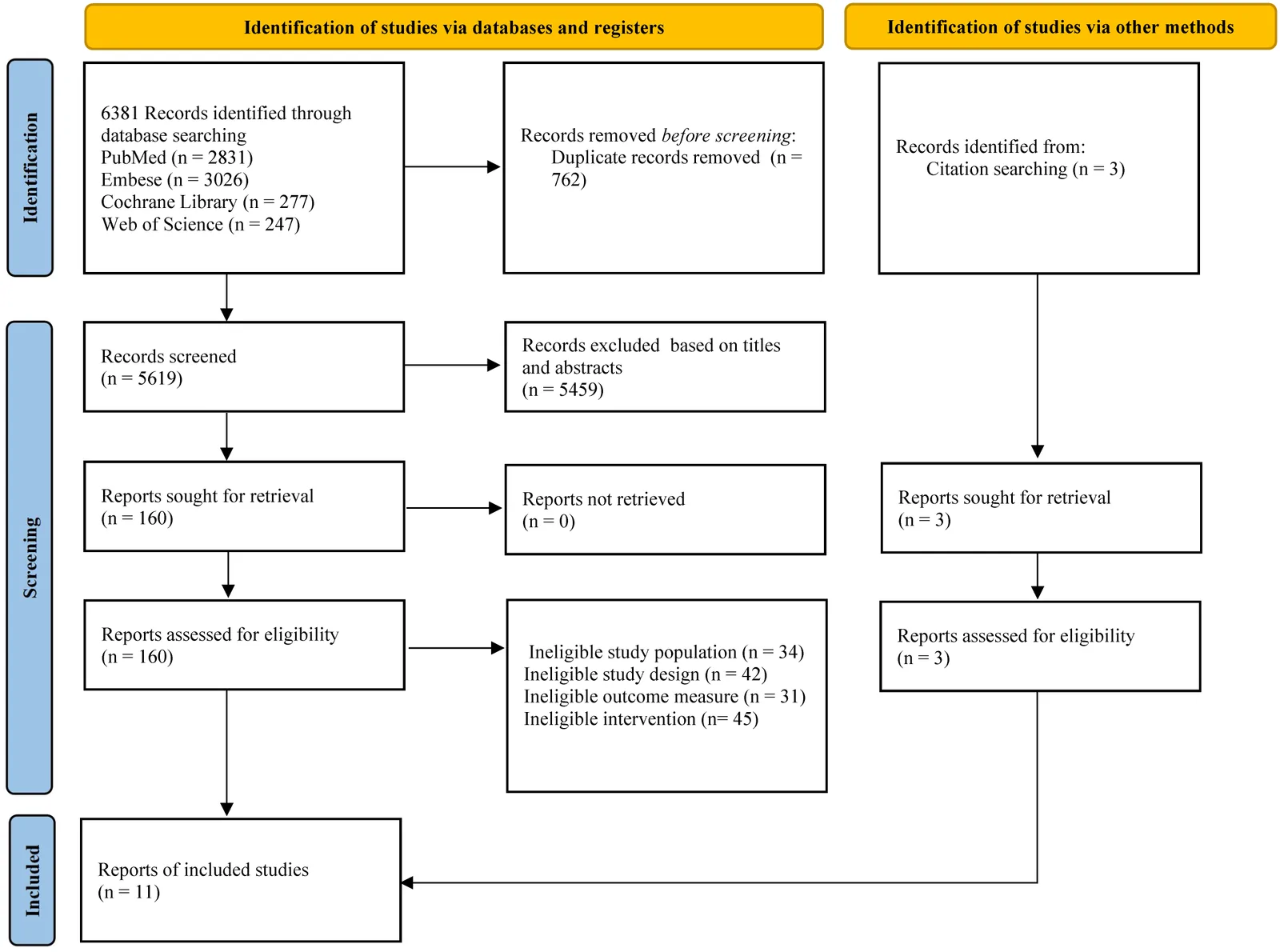
PRISMA flow diagram.

### 3.2 General characteristics of included studies

A total of 11 studies were included. Given that autoimmune encephalitis is a rare neurological disorder, low disease incidence poses challenges for patient recruitment. Moreover, ethical considerations surrounding acute-phase immunotherapy and substantial inter-individual variability in treatment strategies complicate the conduct of high-quality RCTs. Consequently, all included studies were cohort studies.

As summarized in **Table 1**, the 11 studies encompassed 1,359 patients and were published between 2021 and 2025, originating from five countries: China, Germany, the United States, Thailand, and Australia. Seven studies were conducted in China and one in Germany. Anti-NMDAR encephalitis constituted the predominant disease subtype, accounting for 978 cases (72%). Patients with moderate-to-severe disease (baseline mRS ≥3) comprised 1,237 cases (91%), indicating a high severity profile among the included population. Female proportions varied considerably across studies and treatment groups (9.1%–80.0%), higher in NMDAR cohorts and lower in LGI1/CASPR2 cohorts. Seven distinct immunotherapeutic strategies were evaluated, including IVMP, IVIG, IVMP+IVIG, IVMP+PE, IVMP+IVIG+PE, IVMP+IA, and IVMP+EFG. The three most frequently administered regimens were IVMP+IVIG (n=597), IVMP alone (n=292), and IVIG alone (n=228). Clinical phenotypes, prior treatments, and comorbidities in the included cohorts are summarized in **Supplementary Table S3**.

**Table 1:** General characteristics of the included studies.

Study: first author, year	Country	Study design	Study population	Treatment	N	Age, median	Female, %	Baseline mRS Score	Outcome measures
Liu 2025 (11)	China	Retrospective	NMDAR	IVMP+EFG IVMP+IVIG IVMP+IA	26 15 23	21 21 26	42.00% 40.00% 61.00%	3.5 [3, 4.75] 3.0 [3, 4] 4.0[3.5;5]	mRS, AEs
Gong 2021 (12)	China	Prospective	NMDAR	IVIG IVMP	112 60	22 25	56.80% 45.10%	4.0[4;5] 4.0[3;5]	mRS, AEs
Rittel 2025 (13)	Germany	Retrospective	NMDAR	IVMP IVMP+IVIG IVMP+PE IVMP+PE+IVIG	31 52 82 57	38 27 34 29	71.00% 73.10% 69.50% 78.90%	3[2;5] 3[1;5] 4[1;5] 5[2;5]	mRS
			LGI1/CASPR2	IVMP IVMP+IVIG IVMP+PE IVMP+PE+IVIG	44 31 46 15	63 62 66 56	31.80% 35.50% 45.70% 53.30%	3[2;5] 3[1;5] 4[1;5] 5[2;5]	mRS
Cai 2022 (14)	China	Retrospective	AE	IVIG IVMP IVIG+IVMP	11 11 56	46 31 40	36.40% 9.10% 44.60%	2.0[1;3] 2.0[1;3] 2.0[1;3]	mRS, AEs
Rodriguez 2021 (15)	United States	Retrospective	LGI1	IVMP IVIG	49 21	66 66	24.00% 33.00%	3[0;5] 3[2;4]	mRS, AEs
Wang 2021 (16)	China	Retrospective	NMDAR	IVIG IVIG+IVMP	84 160	24 26	54.76% 55.00%	4[3;5] 4[3;5]	mRS, AEs
Zhang 2021 (17)	China	Prospective	AE	IVMP+IVIG IVMP+IVIG+PE	24 33	30 25	46.00% 48.00%	5[4;5] 5[4;5]	mRS
Tang 2025 (18)	China	Retrospective	NMDAR	IVMP+IVIG IVMP+IA	30 23	32 21	66.67% 65.22%	4[3;5] 5[4;5]	mRS, AEs
Surawattanawong 2025 (19)	Thailand	Retrospective +Prospective	AE	IVMP+IVIG IVMP+PE	34 10	51 38	67.60% 80.00%	3[2;3] 2[1;2]	mRS, AEs
Bi 2025 (20)	China	Retrospective	AE	IVMP+EFG IVMP IVMP+IVIG	22 55 45	42 38 41	50.00% 32.70% 53.30%	4.0 [3,;4] 3.0 [3,;4] 4.0 [3,;4]	mRS
Seery 2025 (21)	Australia	Retrospective	LGI1	IVMP IVMP+IVIG IVMP+PE	42 46 9	62 62 62	47.00% 47.00% 47.00%	3.0[2;4] 3.0[2;4] 3.0[2;4]	AEs

AE, autoimmune encephalitis (mixed antibody subtypes); NMDAR, anti–N-methyl-D-aspartate receptor; LGI1, anti–leucine-rich glioma-inactivated protein 1; CASPR2, anti–Contactin-associated protein-like 2; IVMP, intravenous methylprednisolone; IVIG, intravenous immunoglobulin; PE, plasma exchange; EFG, efgartigimod; IA, immunoadsorption; AEs, adverse events.

### 3.3 Risk of bias assessment

Two prospective cohort studies (Gong 2021; Zhang 2021) and one combined retrospective–prospective cohort study (Surawattanawong 2025) were rated as good quality. Eight retrospective studies (including Liu 2025, Tang 2025, and Cai 2022) were classified as fair quality, with common limitations including lack of blinded outcome assessment and, in some studies, insufficient control of key confounders (e.g., time from symptom onset to treatment). Overall, the quality of evidence was considered moderate (**Supplementary Table S4**).

### 3.4 Summary of findings

#### 3.4.1 Efficacy outcomes

Regarding the effectiveness of acute-phase therapy, ten studies involving 1,262 patients were eligible, encompassing seven interventions (IVMP monotherapy, IVIG monotherapy, IVMP+IVIG, IVMP+PE, IVMP+IVIG+PE, IVMP+IA, and IVMP+EFG). These formed 15 direct comparison pathways. Changes in the mRS score at 1 month (4 weeks) post-treatment or at hospital discharge were selected as the primary efficacy endpoint, yielding a network with closed loops (**Figure 2A**). Node-splitting analyses demonstrated no significant inconsistency between direct and indirect evidence (P > 0.05) (**Supplementary Figure S1**–**S3**).

**Figure 2:**
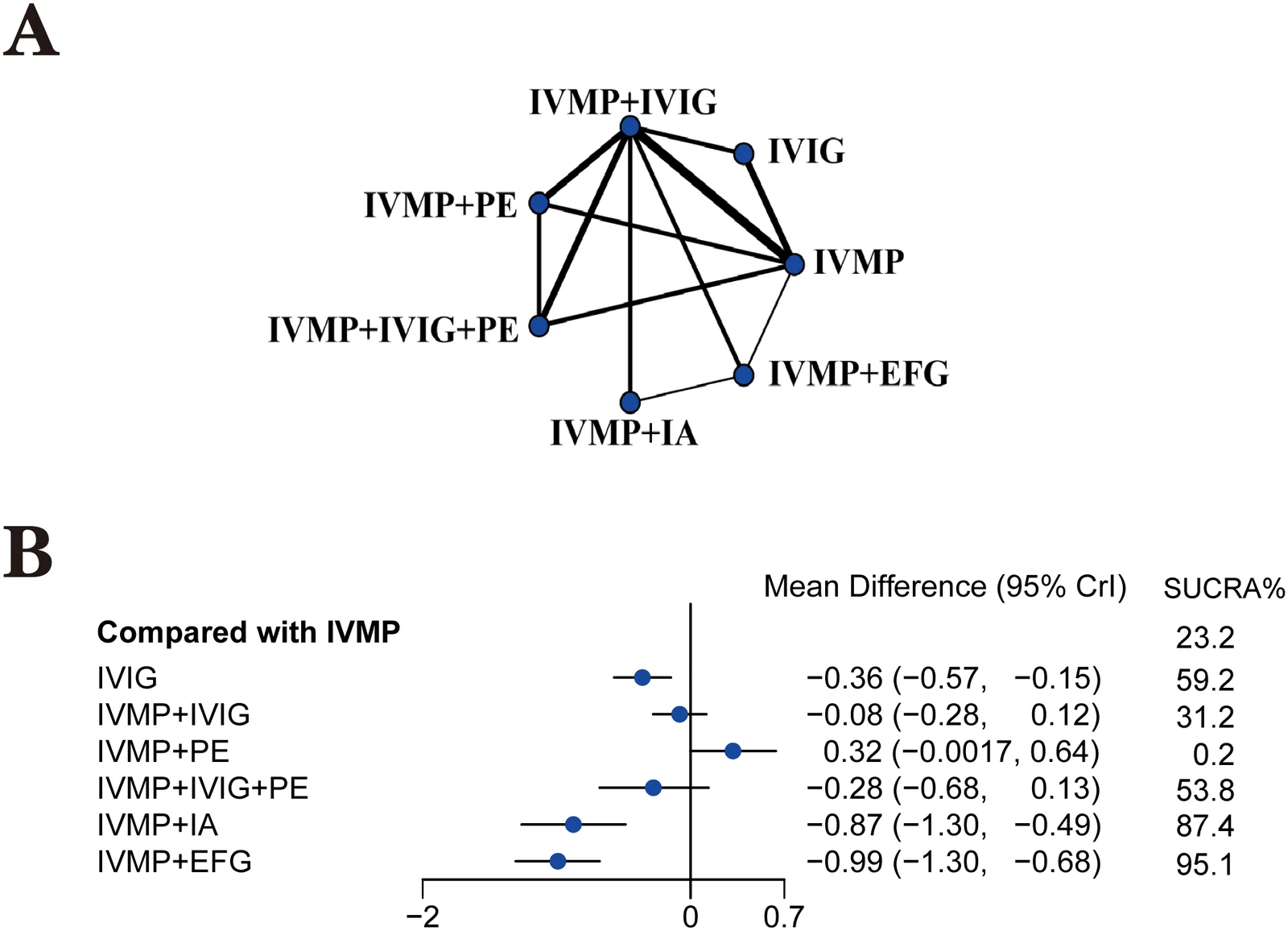
**(A)** Network plot of efficacy in the overall population; **(B)** Forest plot of efficacy in the overall population.

##### 3.4.1.1 Overall population

In the overall population (**Figure 2B**), IVMP+EFG ranked highest in terms of improvement in neurological functional outcomes compared with IVMP monotherapy (MD = −0.99, 95% CrI: −1.30 to −0.68), followed by IVMP+IA (MD = −0.87, 95% CrI: −1.30 to −0.49). By contrast, IVMP+PE exhibited inferior efficacy (MD = 0.32, 95% CrI: −0.0017 to 0.64) without statistical significance. SUCRA rankings further supported these findings, with IVMP+EFG ranking highest (SUCRA = 95.1%), followed by IVMP+IA (SUCRA = 87.4%), whereas IVMP+PE ranked lowest (SUCRA = 0.2%). League table pairwise comparisons indicated no significant difference between IVMP+EFG and IVMP+IA (MD = −0.12, 95% CrI: −0.54 to 0.31). Notably, IVIG monotherapy outperformed IVMP+IVIG (MD = −0.28, 95% CrI: −0.53 to −0.03), suggesting that conventional combination therapy did not confer the anticipated synergistic benefit (**Supplementary Table S5**).

##### 3.4.1.2 Anti-NMDAR encephalitis subgroup

Sensitivity analyses restricted to patients with anti-NMDAR encephalitis (**Figure 3A**) yielded results highly concordant with those of the overall cohort. IVMP+EFG (MD = −0.98, 95% CrI: −1.30 to −0.65) and IVMP+IA (MD = −0.85, 95% CrI: −1.30 to −0.45) remained the more effective regimens, with unchanged SUCRA rankings (IVMP+EFG, 95.0%; IVMP+IA, 88.0%), followed by the remaining interventions.

**Figure 3:**
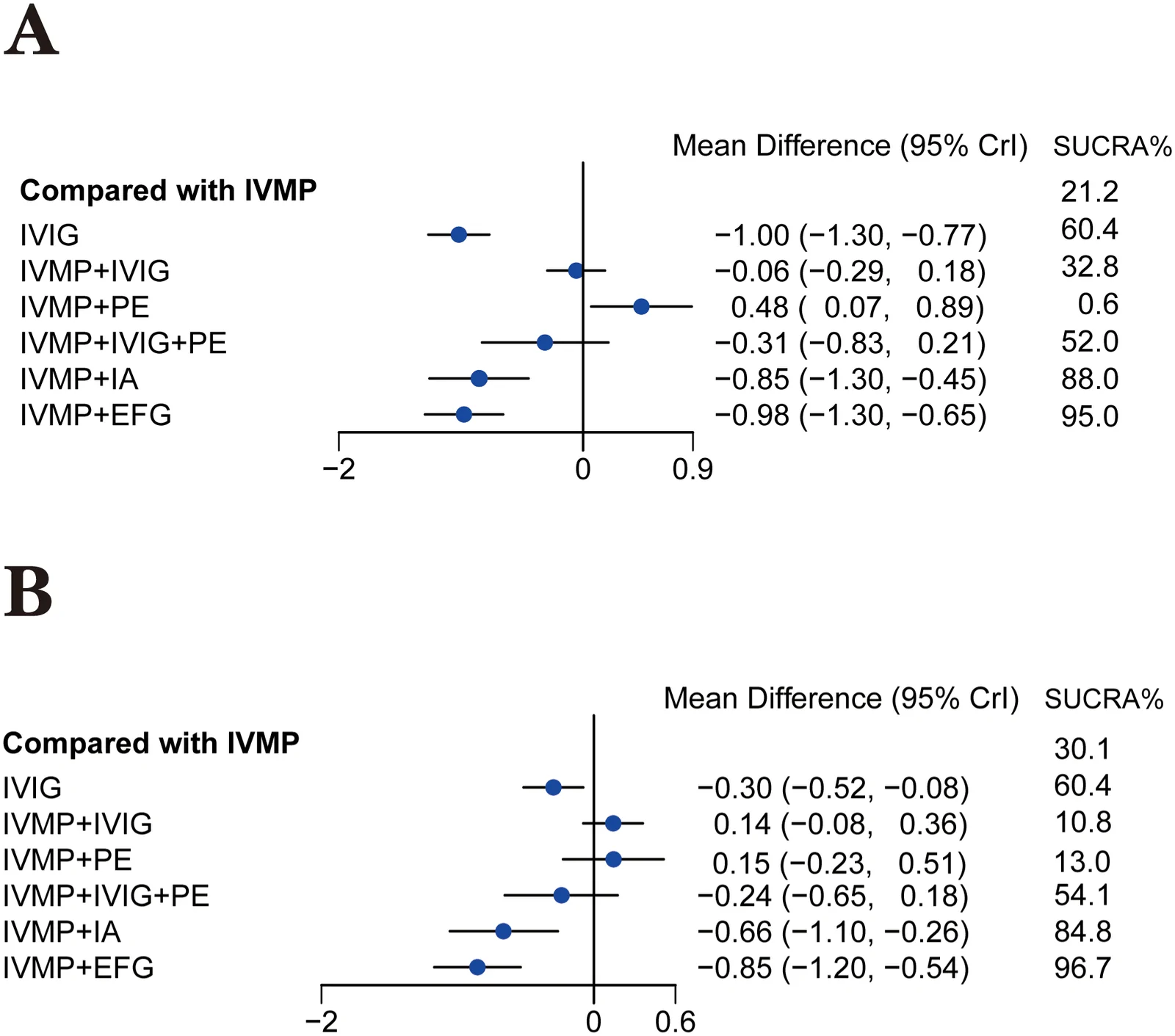
**(A)** Forest plot for Anti-NMDAR encephalitis subgroup; **(B)** Forest plot for the moderate-to-severe subgroup (baseline mRS ≥3).

##### 3.4.1.3 Moderate-to-severe subgroup (baseline mRS ≥3)

In the subgroup of patients with moderate-to-severe disease (baseline mRS ≥3; **Figure 3B**), the therapeutic advantage of IVMP+EFG was further accentuated (MD = −0.85, 95% CrI: −1.20 to −0.54; SUCRA = 96.7%), exceeding its ranking in the overall population. IVMP+IA remained the second most effective option (MD = −0.66, 95% CrI: −1.10 to −0.26; SUCRA = 84.8%). IVIG monotherapy continued to demonstrate a modest benefit (MD = −0.30, 95% CrI: −0.52 to −0.08; SUCRA = 60.4%). In contrast, IVMP+IVIG (MD = 0.14, 95% CrI: −0.08 to 0.36) and IVMP+PE (MD = 0.15, 95% CrI: −0.23 to 0.51; SUCRA = 13.0%) shifted toward potential ineffectiveness, ranking sixth (SUCRA = 10.8%) and fifth (SUCRA = 13.0%). The relative efficacy of each treatment regimen in the overall population and different subgroups is presented in **Supplementary Table S5**–**S7**. Sensitivity analysis excluding the mixed LGI1/CASPR2 cohort replicated the primary findings: IVMP+EFG and IVMP+IA remained superior, while IVMP+PE resulted in less mRS improvement than IVMP in this sensitivity analysis (MD = 0.47, 95% CrI 0.07–0.87; **Supplementary Figure S4**).

#### 3.4.2 Safety outcomes: risk of adverse events

Safety analyses of acute-phase therapy incorporated eight studies comprising 822 patients and six interventions (IVMP monotherapy, IVIG monotherapy, IVMP+IVIG, IVMP+PE, IVMP+IA, and IVMP+EFG). Eight direct comparison pathways were identified, and the incidence of overall adverse events served as the primary safety endpoint, generating a network with closed loops (**Figure 4A**). Node-splitting analyses again revealed no significant inconsistency between direct and indirect comparisons (P > 0.05) (**Supplementary Figure S5**).

**Figure 4:**
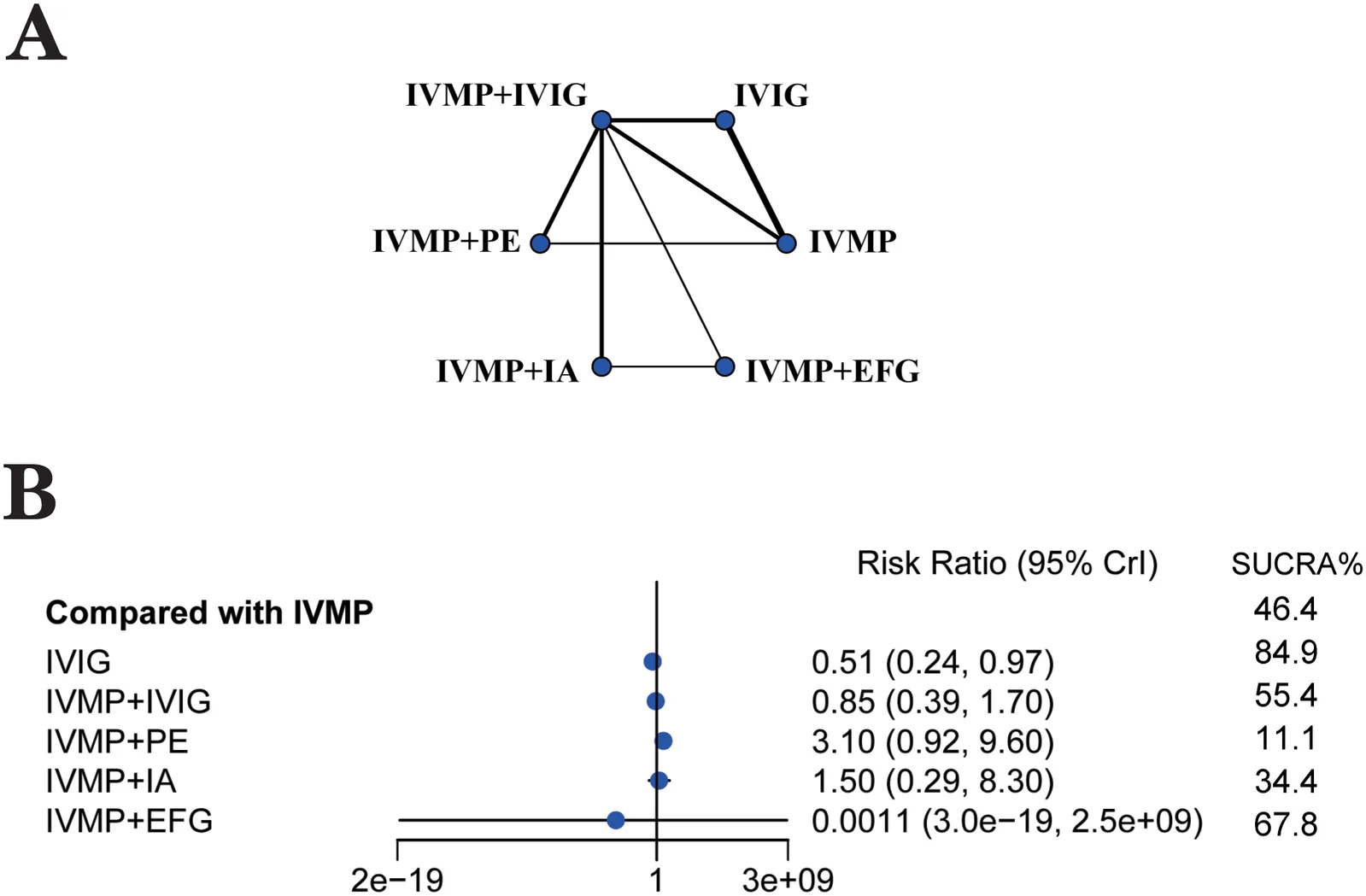
**(A)** Network plot of safety in the overall population; **(B)** Forest plot of safety in the overall population.

Compared with IVMP monotherapy, IVIG monotherapy was associated with the lowest risk of adverse events (RR = 0.51, 95% CrI: 0.24 to 0.97). Conversely, IVMP+PE exhibited the highest risk (RR = 3.10, 95% CrI: 0.92 to 9.60). For IVMP+EFG, the safety estimate was not reliably estimable due to zero-event sparsity (RR = 0.0011, with an extremely wide 95% CrI); accordingly, this intervention was excluded from the safety SUCRA ranking (**Figure 4B**).

## 4 Discussion

Using a Bayesian network meta-analysis, this study comprehensively compared the short-term efficacy and safety of seven immunotherapeutic strategies for acute-phase AE. Several key findings emerged. In terms of efficacy, IVMP+EFG ranked highest for improvement in this analysis in 1-month functional outcomes, with IVMP+IA ranking second; no statistically significant difference was observed between these two regimens. IVIG monotherapy yielded superior short-term functional improvement than IVMP+IVIG, and no significant efficacy difference was detected between IVMP+IVIG and IVMP alone, indicating that conventional combination therapy did not show the expected synergistic effect in this analysis. These conclusions were consistently reproduced in both the anti-NMDAR encephalitis subgroup and the moderate-to-severe disease subgroup, thereby offering supportive evidence for individualized therapeutic decision-making. From a safety perspective, IVIG monotherapy was associated with the lowest risk of adverse events, whereas IVMP+PE carried the highest risk and demonstrated suboptimal efficacy.

### 4.1 IVMP+EFG

In this analysis, IVMP+EFG exhibited favorable clinical efficacy in the acute-phase treatment of AE patients, consistent with the findings reported by Liu et al. and Bi et al. This therapeutic benefit is tied to the complex pathophysiology of AE. Central to AE pathogenesis is the production of autoantibodies targeting neuronal surface or intracellular antigens, which trigger downstream immune responses, leading to aberrant attacks on the central nervous system and ultimately causing neuronal injury or functional impairment (22). IVMP mitigates disease activity by inhibiting pro-inflammatory signaling pathways such as NF-κB, thereby reducing cytokine release and suppressing B-cell differentiation and autoantibody production (23); additionally, IVMP transiently increases blood-brain barrier permeability, facilitating subsequent drug penetration into the CNS (24). EFG, an FcRn antagonist, specifically blocks IgG recycling, markedly shortening the half-life of pathogenic IgG antibodies and accelerating their clearance. Importantly, EFG selectively targets pathogenic IgG and spares protective immunoglobulins, highlighting its precision-targeted therapeutic advantage (25). Furthermore, the combination of IVMP and EFG may exert synergistic effects. IVMP suppresses inflammatory responses and antibody production at the source, whereas EFG accelerates the clearance of pathogenic antibodies from the circulation. With complementary mechanisms, the two agents can more rapidly reduce the pathogenic antibody burden *in vivo* and improve clinical symptoms (26).

### 4.2 IVMP+IA

IVMP+IA ranked second in efficacy among the evaluated regimens, with efficacy primarily driven by direct and efficient antibody removal. IA utilizes specific ligands (e.g., tryptophan, protein A) to mediate antigen-antibody binding or leverage physicochemical interactions, thereby directly depleting circulating autoantibodies and immune complexes. This process interrupts ongoing attacks on neuronal synapses, diminishes complement activation, and lowers complement-dependent cytotoxicity. Such findings align with prior studies linking antibody clearance efficiency to clinical improvement (27, 28). Recent evidence indicates that in AE subtypes such as VGKC complex-associated encephalitis, IA outperforms conventional PE in antibody clearance (29), positioning it as a rapid and primary antibody-depleting modality within combination regimens. In IVMP+IA therapy, IVMP provides background immunosuppression by reducing *de novo* antibody production (30), while IA directly eliminates circulating antibodies. This dual mechanism enables a faster and more complete reduction of antibody titers, enhancing clinical efficacy.

### 4.3 IVMP+PE

In this study, IVMP+PE showed suboptimal efficacy and a higher adverse-event risk. Most patients had anti-NMDAR encephalitis, in which pathogenic IgG1 antibodies may have substantial intrathecal involvement. PE only removes only 60–70% of circulating IgG and is less effective against intrathecal antibodies (13), which may limit its benefit in this population. In addition, PE requires vascular access and anticoagulation, while high-dose IVMP may increase susceptibility to infection and induce transient hypercoagulable (31–33). These overlapping mechanisms may worsen the overall risk–benefit profile of IVMP+PE.

### 4.4 IVMP+IVIG

IVIG monotherapy outperformed IVMP+IVIG in this analysis, whereas no significant efficacy difference was observed between IVMP+IVIG and IVMP alone. This may be partly explained by overlapping immunomodulatory mechanisms: IVMP broadly suppresses immune activation (34), while IVIG modulates autoimmune responses through pathogenic antibody neutralization, Fc receptor blockade, complement regulation, regulatory T-cell modulation, and inhibition of NF-κB activation (35, 36). Since IVIG itself inhibits NF-κB activation, combining it with IVMP may not produce fully additive therapeutic effects and may even compound immunosuppression. Consistently, Cai 2022 (14) reported no significant short-term efficacy difference between IVIG monotherapy and IVMP+IVIG, and Wang 2021 (16) found relatively poorer short-term outcomes with IVMP+IVIG in anti-NMDAR encephalitis, partly related to heightened inflammatory response and increased nosocomial infection risk. These findings highlight the need for individualized use of IVMP+IVIG rather than assuming superiority over monotherapy.

### 4.5 Safety profiles of acute-phase immunotherapies

In terms of safety, the risk of adverse events differed notably across treatment regimens. IVIG monotherapy demonstrated the lowest adverse-event risk in this analysis (RR = 0.51, 95% CrI: 0.24 to 0.97), consistent with Gong 2021 (12). Mechanistically, IVIG acts as a natural immunomodulator via Fc receptor saturation, neutralization of pathogenic IgG1 antibodies, complement regulation, regulatory T-cell modulation, and IL-10-mediated monocyte-macrophage polarization toward an anti-inflammatory phenotype (37, 38). Unlike cytotoxic therapies, it avoids broad immune damage, leading to low infection risk. IVIG-related adverse events are predominantly mild and typically manifest as infusion reactions, headache, fever, nausea, or hyperosmolarity-related discomfort. Conversely, severe complications such as thrombosis, hemolysis, aseptic meningitis, or renal impairment are rare but require careful monitoring in high-risk patients (39, 40).

By contrast, IVMP exerts broad anti-inflammatory and immunosuppressive effects, with corticosteroid-related adverse events including metabolic disturbance, hypertension, gastrointestinal irritation, mood or sleep disturbance, hepatic dysfunction, osteonecrosis, and increased susceptibility to infection (41). Combining it with IVIG or PE may overlap these risks with the other therapy’s side effects. PE removes circulating pathogenic antibodies and inflammatory mediators but also non-selectively depletes coagulation factors, immunoglobulins, plasma proteins, and electrolytes; therefore, PE-containing regimens may increase the risks of hypotension, catheter-related infection, bleeding tendency, electrolyte imbalance, hypoproteinaemia, and thrombotic complications (42). Together with corticosteroid-induced immunosuppression and hypercoagulability, these mechanisms may explain the higher adverse-event risk observed for IVMP+PE in this study (43). IA may be more selective than PE for antibody removal but still carries risks like vascular access issues or haemodynamic complications (44). For EFG, potential adverse events include mild-to-moderate infections, headache, and infusion-related reactions (45); in this network, IVMP+EFG safety was not reliably estimable due to zero events and excluded from ranking.

### 4.6 Clinical implications and treatment decision pathways

Based on these findings, a stratified, individualized approach is recommended when selecting acute-phase immunotherapy for adult AE patients. For moderate-to-severe cases (baseline mRS ≥3), particularly those with anti-NMDAR encephalitis, IVMP+EFG and IVMP+IA may be considered potential treatment options, but the current evidence is insufficient to establish their clinical priority over conventional regimens. For patients prioritizing safety, for those with contraindications to corticosteroids, or in settings lacking access to novel agents, IVIG monotherapy constitutes a rational clinical approach, underpinned by its favorable safety profile. The analysis also suggests that traditional combination therapy with IVMP+IVIG did not confer clear synergistic efficacy over monotherapy, whereas IVMP+PE exhibited limited efficacy coupled with a higher risk of adverse events. Clinical application of these regimens should therefore involve careful assessment of risk–benefit ratios. Future large-scale, prospective studies are warranted to further evaluate the long-term efficacy, safety, and cost-effectiveness of these strategies to optimize acute-phase AE management.

### 4.7 Strengths and limitations of this study

This study offers significant academic and clinical value, with key strengths in two domains: First, Comprehensiveness and novelty. This study represents the first systematic comparison of seven acute-phase immunotherapies, including novel interventions such as EFG and IA, and addresses controversies regarding the synergistic effects of traditional combination therapies. This fills a gap in prior research that lacked comprehensive evaluation of new treatment modalities and combined regimens. Second, clinical applicability. Subgroup analyses of moderate-to-severe patients and anti-NMDAR encephalitis cases clarified patient populations more likely to benefit from each intervention. Integration of pathophysiological mechanisms provided plausible explanations for observed efficacy differences among subgroups, enhancing translational relevance. Nevertheless, several limitations should be acknowledged. First, reporting standards for baseline characteristics and outcome measures were not uniform across studies. Neuropsychiatric symptoms, particularly memory impairment, may have been systematically underreported, as prominent acute-phase manifestations could mask cognitive deficits and introduce bias. Data on autoantibody titers and CASE scores were scarce and heterogeneous in their assessment criteria, precluding stable quantitative synthesis in a network meta-analysis (**Supplementary Table S8**, **S9**). Moreover, most original studies did not provide adjusted effect estimates; our pooled analyses were therefore based primarily on unadjusted data, with insufficient control for confounding factors. Consequently, the treatment rankings should be interpreted as exploratory comparative findings requiring confirmation in prospective studies. In addition, most included studies were observational in design, and follow-up durations varied, which may have further introduced selection bias. Second, the generalizability of our findings is limited, and evidence for certain subgroups remains weak. Given that the study cohort was predominantly Asian, extrapolating these conclusions to other ethnic populations or distinct healthcare settings requires careful consideration. ICI-treated patients were excluded due to their neurological AEs’ distinct pathophysiology and specific management needs. ICI-associated encephalitis represents a distinct treatment-related neuroimmune condition. Management commonly involves withholding the causative ICI and initiating corticosteroids, while IVIG, plasma exchange, or additional immunosuppression may be considered in severe or steroid-refractory cases (46). Treatment responses in this setting may therefore differ from those observed in conventional neuronal surface antibody-mediated AE. Classical PNS were not prioritized; accordingly, our findings should not be extrapolated to these populations. Unbalanced distribution of sex and antibody subtypes across studies and treatment groups, and the lack of individual-level stratified data precluded reliable sex-adjusted analyses. Data on CASPR2 antibody-associated cohorts were limited, and most original studies did not report outcomes separately for this subgroup, preventing independent conclusions regarding optimal treatment strategies. Furthermore, small sample sizes for some novel regimens, particularly IVMP+EFG, may compromise the precision of effect estimates; the wide credible intervals for safety outcomes underscore the need for larger-scale confirmatory studies. Third, substantial heterogeneity existed in treatment protocols across studies. IVMP dosage and duration, oral prednisone tapering regimens, IVIG infusion schedules, and the frequency and interval of PE or IA sessions varied considerably. These parameters may influence the intensity of immunosuppression, the efficiency of autoantibody clearance, and the risk of adverse events, thereby exacerbating inter-study clinical heterogeneity and diminishing the robustness of the pooled network estimates. Fourth, the definitions and recording standards for adverse events were inconsistent across studies. The available data permitted only the aggregation of patients experiencing at least one adverse event per treatment group, precluding further stratification by severity or specific event type. Therefore, our safety findings should be interpreted as reflecting overall adverse event risk rather than the incidence for particular categories of adverse events.

In summary, standardized reporting of baseline characteristics, treatment protocols, autoantibody titers, disease-specific scale scores, and adverse event outcomes is recommended for future studies. Additionally, large-scale, multicenter prospective investigations are needed to validate current findings and enhance the comparability of evidence across different immunotherapeutic strategies.

## 5 Conclusion

In the acute-phase management of AE, IVMP+EFG and IVMP+IA ranked highest in efficacy in the current network meta-analysis, particularly in moderate-to-severe patients (baseline mRS ≥3) and those with anti-NMDAR encephalitis. Regarding safety, IVIG exhibited the lowest risk of adverse events. Future investigations should prioritize multicenter, large-scale, prospective RCTs to validate the long-term efficacy and cost-effectiveness of novel immunotherapeutic strategies in AE.

## Data Availability

The original contributions presented in the study are included in the article/**Supplementary Material**. Further inquiries can be directed to the corresponding author.
