# Supplementary material for: Efficacy and safety of acute-phase immunotherapy for adult autoimmune encephalitis: a systematic review and network meta-analysis
Source: Front Immunol. 2026 Oct 7;17:1807436. doi: 10.3389/fimmu.2026.1807436 (PMC13683221; doi:10.3389/fimmu.2026.1807436)
Supplement: Supplementary file 1 [file SupplementaryFile1.docx]

**Figure S1. FTO expression in lung tissues from experimental rat groups.** (A), FTO mRNA levels across groups, measured by qRT-PCR. (B), Representative western blot images of FTO protein expression in lung tissue lysates. c, Semiquantitative analysis of FTO protein levels from (b). Data are presented as mean ± s.d. (n = 5-8 rats per group). Statistical significance was determined by one-way ANOVA followed by Tukey's post hoc test. ns, not significant (*p* > 0.05); *****p* < 0.0001.

**Figure S2. Validation of FTO overexpression and knockdown efficiencies in RLE-6TN cells.** (A-C), FTO levels in the overexpression model. (A)**,** mRNA levels measured by qRT-PCR. (B)**,** Representative western blot of FTO protein. (C)**,** Quantification of FTO protein expression from B. (D-F), FTO levels in the knockdown model. (D), mRNA levels measured by qRT-PCR. (E), Representative western blot of FTO protein. (F), Quantification of FTO protein expression from E. Data are presented as mean ± s.d. (n=3 independent experiments). Statistical significance was determined by one-way ANOVA with Tukey’s post hoc test. ns, not significant (*p*>0.05); **p*<0.05; ***p*<0.01; ****p*<0.001; *****P*<0.0001. Abbreviations: WT, wild-type; Vector, empty vector control; OE-FTO, FTO-overexpressing; LV-NC, non-targeting shRNA control; LV-FTO#1/2/3, FTO-targeting shRNA (sites #1, #2, #3).

**Figure S3. Validation of PRKCB overexpression and knockdown efficiencies in RLE-6TN cells.** (A-C), PRKCB levels following overexpression. (A), mRNA levels by qRT-PCR. (B), Representative western blot. (C), Quantification of protein expression from (B). (D-F), PRKCB levels following knockdown. (D), mRNA levels by qRT-PCR. (E), Representative western blot. (F), Quantification of protein expression from (E). Data are presented as mean ± s.d. (n=3 independent experiments). Statistical significance was determined by one-way ANOVA with Tukey's post hoc test. ns, not significant (*p*>0.05); **p<0.05*; ***p*<0.01; ****p*<0.001; *****p*<0.0001. Abbreviations: WT, wild type; Vector, empty vector control; OE-PRKCB, PRKCB-overexpressing; LV-NC, non-targeting shRNA control; LV-PRKCB#1/2/3, PRKCB-targeting shRNA (sites #1, #2, #3).
